# Transcriptome Sequencing Reveals the Virulence and Environmental Genetic Programs of *Vibrio vulnificus* Exposed to Host and Estuarine Conditions

**DOI:** 10.1371/journal.pone.0114376

**Published:** 2014-12-09

**Authors:** Tiffany C. Williams, Elliot R. Blackman, Shatavia S. Morrison, Cynthia J. Gibas, James D. Oliver

**Affiliations:** 1 Department of Biological Sciences, University of North Carolina at Charlotte, Charlotte, North Carolina, United States of America; 2 Department of Bioinformatics and Genomics, University of North Carolina at Charlotte, Charlotte, North Carolina, United States of America; The Scripps Research Institute and Sorrento Therapeutics, Inc., United States of America

## Abstract

*Vibrio vulnificus* is a natural inhabitant of estuarine waters worldwide and is of medical relevance due to its ability to cause grievous wound infections and/or fatal septicemia. Genetic polymorphisms within the virulence-correlated gene (*vcg*) serve as a primary feature to distinguish clinical (C-) genotypes from environmental (E-) genotypes. C-genotypes demonstrate superior survival in human serum relative to E-genotypes, and genome comparisons have allowed for the identification of several putative virulence factors that could potentially aid C-genotypes in disease progression. We used RNA sequencing to analyze the transcriptome of C-genotypes exposed to human serum relative to seawater, which revealed two divergent genetic programs under these two conditions. In human serum, cells displayed a distinct “virulence profile” in which a number of putative virulence factors were upregulated, including genes involved in intracellular signaling, substrate binding and transport, toxin and exoenzyme production, and the heat shock response. Conversely, the “environmental profile” exhibited by cells in seawater revealed upregulation of transcription factors such as *rpoS, rpoN*, and *iscR*, as well as genes involved in intracellular signaling, chemotaxis, adherence, and biofilm formation. This dichotomous genetic switch appears to be largely governed by cyclic-di-GMP signaling, and remarkably resembles the dual life-style of *V. cholerae* as it transitions from host to environment. Furthermore, we found a “general stress response” module, known as the stressosome, to be upregulated in seawater. This signaling system has been well characterized in Gram-positive bacteria, however its role in *V. vulnificus* is not clear. We examined temporal gene expression patterns of the stressosome and found it to be upregulated in natural estuarine waters indicating that this system plays a role in sensing and responding to the environment. This study advances our understanding of gene regulation in *V. vulnificus*, and brings to the forefront a number of previously overlooked genetic networks.

## Introduction

*Vibrio vulnificus* is a free-living inhabitant of estuarine and coastal waters world-wide, and associates with a variety of aquatic organisms [1]. This bacterium is also a highly invasive pathogen of both fish and humans, and is the primary cause of sea-food related deaths in the US, typically from ingestion of raw or undercooked molluscan shellfish [2]. This medically and economically relevant organism can cause rapidly fulminating septicemia when ingested. If able to gain entry through an open cut or wound, this pathogen can also cause necrotizing fasciitis, often resulting in limb amputation, and can lead to fatal septicemia in susceptible individuals [3].

Not all strains of *V. vulnificus* are equally pathogenic, thus strains are grouped into biotypes and genotypes. Strains within biotype 1 represents those most often associated with disease in humans, whereas biotype 2 represents strains are almost exclusively associated with disease in eels [4]. The most recently discovered biotype 3 consists of strains which are genetically distinct from biotypes 1 and 2, a pathogen which to date is geographically limited to Israel [5], [6]. A PCR-based assay can be used to separate *V. vulnificus* biotype 1 strains into two groups that strongly correlate with source of isolation, such that “environmental isolates” possess the *vcgE* allele, whereas “clinical isolates” have the *vcgC* allele [7], [8]. Thus, we further subtype biotype 1 strains into two groups: clinical (C-genotypes) and environmental (E-genotypes). Multilocus sequence typing and phylogenetic analyses of conserved housekeeping loci and putative virulence loci have further substantiated the observed dimorphism between *V. vulnificus* biotype 1 strains [9], [10]. Additionally, a phylogenetic analysis of 175 genes present in all currently sequenced *Vibrio* species revealed the same trend, with all six *V. vulnificus* strains examined grouping into one clade, although a distinct branching between C- and E-genotypes was observed [11]. These results indicate that the C/E differences observed are not restricted to a few loci, but are genome-wide and led to the proposition that these two genotypes represent distinct ecotypes [9].

Undoubtedly, *V. vulnificus* C- and E-genotypes display significant differences in their ecology, physiology, genome content, and genetic responses. C-genotypes can resist the bactericidal effects of human serum and even grow in this environment, whereas E-genotypes largely succumb to these bactericidal effects and rapidly decline in number shortly after exposure [12]. Indeed, human serum has become a popular model for predicting virulence amongst environmentally isolated strains [13], [14]. This correlation between genotype and virulence has been further substantiated in the mammalian model of disease, where C-genotypes are more likely to cause systemic infection and death relative to E-genotypes [15]. Furthermore, a recent comparative genomic analysis of three C- and three E-genotype strains revealed that while these share approximately 3664 genes, they also possess genes unique to each genotype. Of the 278 genes unique to C-genotypes, many were found to represent putative virulence factors, whereas 167 E-specific genes were associated with metabolic functions and may have implications for nutritional competence [11]. Nevertheless, the elusive question of which specific genetic features contribute to the observed differences in environmental distribution and pathogenic potential still stands.

The goal of the current study was to analyze the transcriptome profile of two clinically isolated *V. vulnificus* C-genotypes exposed to human serum (HS) or artificial seawater (ASW). Using RNA sequencing, we screened the transcriptome for clinically relevant genes (or sets of genes), to provide a snapshot of the gene content within clinical strains under these two conditions. We identified several genetic features that likely contribute to survival in the natural aquatic environment as well as the host environment, many of which are relatable to one another. Additionally we found several differentially expressed genes to be unique to C-genotypes and lacking in E-genotypes. From a holistic perspective, our results indicate that in ASW cells take on a low virulence, enhanced biofilm phenotype which we refer to as the “environmental profile”, whereas in HS, cells exhibit a “virulence profile” in which biofilm formation is inhibited and virulence factor production predominates. Notably, this dichotomy in genetic programming between HS and ASW remarkably resembles the genetic and phenotypic switch documented in *V. cholerae* cells as they transition from host to environment. Here, we highlight some of these differentially expressed genes, and discuss the potential relevance of each gene set, thereby setting the stage from which future studies can be directed.

## Results and Discussion

### RNA sequencing results

**Figure 1:**
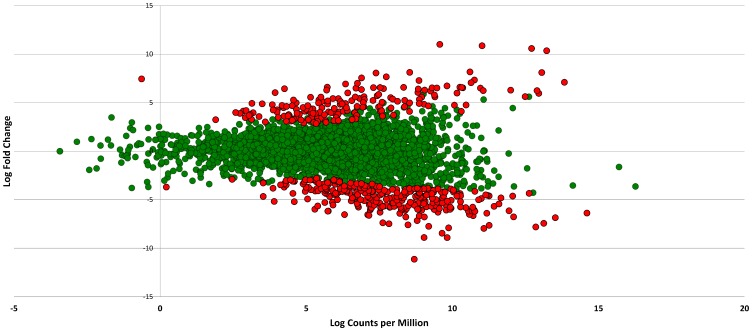
Smear plot of differentially expressed genes in *V. vulnificus* CMCP6 exposed to human serum (relative to artificial seawater). The smear plot shows the relationship between the log fold change and log counts per million. Green points represent non-significant DE genes whereas red points show genes that are significantly differentially expressed (p<0.0001) in relation to artificial seawater.

**Figure 2:**
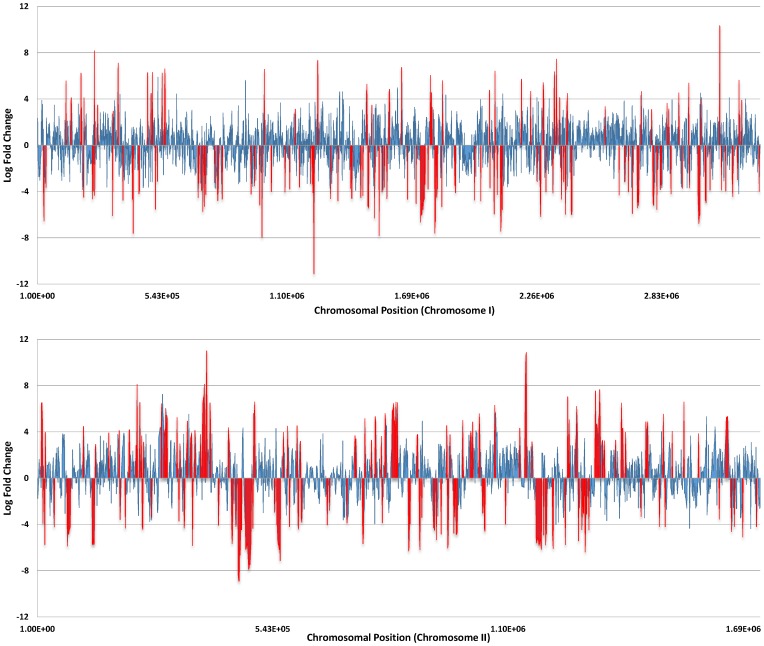
Linear compressed view of differentially expressed genes in *V. vulnificus* exposed to human serum (relative to artificial seawater). These charts show a compressed view of the differentially expressed genes in *V. vulnificus* CMCP6 by chromosome (top; chromosome I, bottom; chromosome II) and allows quick identification of clusters of differentially expressed genes, both positive and negative. The y-axis shows the log fold change and the x-axis is the nucleotide position of the chromosome. Blue bars represent non-significant DE genes whereas red bars show genes that are significantly differentially expressed (p<0.0001) in relation to artificial seawater.

Two blood isolates of *V. vulnificus* (CMCP6 and YJ016) were exposed to human serum (HS) or 10ppt artificial seawater (ASW), and cDNA prepared from mRNA isolated from each strain was subjected to Illumina sequencing. Comparative transcriptome analysis of *V. vulnificus* cells exposed to human serum relative to artificial seawater resulted in a total of 469 and 653 differentially expressed (DE) genes (p-value <0.0001) in CMCP6 and YJ016, respectively (**Table 1**). **Fig. 1** depicts a smear plot of DE genes in CMCP6 with a distinction between those transcripts deemed to be significantly DE (**S1 Figure** depicts the smear plot for YJ016). **Fig. 2** shows a compressed linear view of DE genes in CMCP6 by chromosome. **S2 Figure** depicts the same information for YJ016. A full list of differentially expressed genes in CMCP6 and YJ016 when grown in HS relative to ASW can be found in **S1 File**. Each file lists the protein number (NP) associated with each gene, respective product descriptions, expression levels (expressed as log fold change), direction of regulation (+ or −), and p-values to indicate level of significance. **S2** **and** **S3 Files** contain organized files of selected DE genes discussed in detail in this study. **S2 File** lists genes upregulated in HS and **S3 File** lists genes upregulated in ASW.

**Table 1:** Summary of differentially expressed genes (p<0.0001).

	CMCP6	YJ016
No. of differentially expressed genes		
Genome-wide	469	653
Chromosome I	220	302
Positively expressed	59	79
Negatively expressed	161	147
Chromosome II	249	347
Positively expressed	120	223
Negatively expressed	129	200
Plasmid	N/A^a^	4
Percent of differentially expressed genes		
Genome-wide	10.3%	12.6%
Chromosome I^b^	7.3%	8.9%
Chromosome II	16%	20.3%
Plasmid	N/A	5.8%

a N/A – not applicable.

b Chromosomal percentages were calculated as number of DE genes divided by total number of genes on each chromosome.

An interesting aspect of our study was the observed imbalance of differentially expressed genes between the two chromosomes of *V. vulnificus*. A higher percentage of genes were upregulated on chromosome II relative to chromosome I. This phenomenon can be easily visualized using a compressed linear view of the differentially expressed genes based on chromosomal position (**Fig. 2** and **S2 Figure**). These charts (assembled using edgeR output, featureCounts output, and Excel) allow for quick identification of differentially expressed genes (depicted in red), both positive and negative.

In *Vibrio* spp., the larger chromosome (I) is relatively constant in size and contains most of the housekeeping genes essential for growth and viability, whereas the smaller chromosome (II) has a tendency to vary in size and contains many unknown or hypothetical genes [16]. Interspecies comparisons of chromosome II reveals a large degree of genomic variation and this diversity has been suggested to play a role in adaptation to environmental changes and may be important for species-specific functions [16]. Our study highlights the utility of chromosome II in facilitating survival in an unnatural environment for *V. vulnificus*.

#### GO term enrichment analysis

Gene Ontology (GO) term enrichment analysis of the differentially expressed genes in the two strains revealed a consistent pattern of functional enrichment. 15 enriched terms were identified for CMCP6 and 16 for YJ016, with the primary differences being the ranking of terms. In the cellular component term hierarchy, which describes the localization of the gene, the only significantly enriched term for both CMCP6 and YJ016 was GO:0016020 (membrane). In the molecular function hierarchy, which describes molecular activities, the only significantly enriched term, again appearing in both species, was GO:0060089 (molecular transducer activity). All remaining significantly enriched terms identified were within the biological process hierarchy, and included such biological roles as cellular response to stimulus, signal transduction, cellular communication, formamide and other amide metabolism, and (in the case of YJ016), taxis (**Table 2**).

**Table 2:** Enriched Gene Ontology Categories of Differentially Expressed Genes in human serum and artificial seawater.

	CMCP6				YJ106			
	Human serum		Seawater		Human serum		Seawater	
Description	No. DE genes	Fold exp. range	No. DE genes	Fold exp. range	No. DE genes	Fold exp. range	No. DE genes	Fold exp. range
Membrane	41	2.8 to 8.1	58	7.6 to 2.8	48	2.7 to 9.8	68	8.1 to 2.6
Response to stimulus	13	2.9 to 7.1	51	7.6 to 3.1	18	3.2 to 7.9	56	6.3 to 2.6
Cellular response	6	2.9 to 6.3	45	7.6 to 3.2	9	3.5 to 5.6	49	6.3 to 2.6
Cellular communication	6	2.9 to 6.3	43	7.6 to 3.2	9	3.5 to 5.6	46	3.6 to 2.6
Molecular transducer activity	6	2.9 to 5.2	37	7.6 to 3.2	9	3.5 to 3.9	39	6.3 to 2.6
Signal Transduction	5	2.9 to 4.8	42	7.6 to 3.2	9	3.5 to 5.6	45	6.3 to 2.6
Signaling	5	2.9 to 4.8	42	7.6 to 3.2	9	3.5 to 5.6	45	6.3 to 2.6
Single organism signaling	5	2.9 to 4.8	42	7.6 to 3.2	9	3.5 to 5.6	45	6.3 to 2.6
Regulation of RNA metabolism	15	2.9 to 6.7	12	7.4 to 3.9	14	2.7 to 7.0	20	8.3 to 2.5
Regulation of macromolecule biosynthesis	15	2.9 to 6.7	12	7.4 to 3.9	14	2.7 to 7.0	20	8.3 to 2.5
Regulation of cell macromolecules	15	2.9 to 6.7	12	7.4 to 3.9	14	2.7 to 7.0	20	8.3 to 2.5
Histidine catabolism	0	NA^a^	4	6.0 to 5.4	0	NA	4	6.0 to 5.4
Imidazole containing compounds	0	NA	4	6.0 to 5.4	0	NA	4	6.0 to 5.4
Formamide metabolism	0	NA	4	6.0 to 5.4	0	NA	4	6.0 to 5.4
Formate metabolism	0	NA	4	6.0 to 5.4	0	NA	4	6.0 to 5.4
Taxis	NA	NA	NA	NA	1	3.5	13	−6.3 to −2.9

a – not applicable.

### Genes induced in human serum

#### Substrate binding and transport

In the human host, *V. vulnificus* must overcome a number of physiological challenges to survive and multiply. *In vivo* growth requires the acquisition of scarce but essential nutrients, such as iron and sulfur, which are critical to cell function. Indeed we identified genes involved in iron acquisition to be upregulated in human serum. Iron is an essential micronutrient for *V. vulnificus*, and is critical for growth and virulence [17], thus this bacterium employs siderophore-based systems to chelate iron from high-affinity binding proteins such as ferritin, transferrin, and lactoferrin. *V. vulnificus* also makes use of an OM receptor, HupA, to acquire iron from heme-containing compounds such as hemoglobin and cytochromes [18]. In our transcriptome analysis, we found the receptor and transport genes for the hydroxamate siderophore, the biosynthetic, transport, and receptor genes for the catechol siderophore (vulnibactin), and hupA to be induced upon exposure to human serum. This finding corroborates a previous study in which *V. vulnificus* expression of *vuuA* and *fhuA* (encoding the vulnibactin and hydroxamate siderophore receptors, respectively), was significantly enhanced in human serum compared to natural seawater [19].

*V. vulnificus* utilizes three TonB systems (TonB1, TonB2, and TonB3) to transport both the hydroxamate-type siderophore and vulnibactin, as well as heme and hemoglobin [20]. The TonB systems consist of three integral inner membrane proteins (TonB, ExbB, and ExbD), which generate the energy needed to activate the OM TonB receptor proteins. The operons coding for TonB1 and TonB2 were found to be significantly upregulated in HS, however TonB3 was not differentially expressed. This finding strongly supports a previous study from the Crosa lab which found *V. vulnificus* TonB1 and TonB2 systems to be induced under iron-limiting conditions whereas TonB3 transcription did not change in response to iron concentrations [21].

Our study found the operon (*potABCD*) encoding spermidine transport to be up-regulated in human serum. Polyamines such as spermidine, are a class of small organic compounds with a hydrocarbon backbone and multiple amino groups, are known to play a crucial role in maintaining optimal conformation of nucleic acids, and are therefore essential for normal cellular growth and multiplication [22]. However, research has unveiled some novel functions of polyamines in microorganisms linking these compounds with biofilm formation, escape from phagolysosomes, bacteriocin production, toxin activity, and protection from oxidative and acid stress [22]. Upregulation of spermidine transport genes in HS indicates that polyamines could serve an important function for *V. vulnificus* within the human host and this proposition deserves further attention.

Clinical isolates of *V. vulnificus* harbor a 33-kb genomic island (region XII) located on chromosome II, which contains an arylsulfatase gene cluster, a sulfate reduction system, two chondroitinases, and an oligopeptide ABC transport system [10]. It has been proposed that acquisition of this genomic island may serve to enhance fitness in the aquatic environment and/or human host, however studies to validate this proposition for *V. vulnificus* have yet to be reported. We found a subset of genes within the genomic region XII to be upregulated in human serum, particularly those within the arysulfatase gene cluster. Arysulfatases hydrolyze arylsulfate ester bonds to release free sulfate, which is essential for bacterial growth and survival [10], [23]. These are typically expressed under conditions of sulfur starvation, but have also been shown to be regulated by monoamine compounds such as norepinephrine, which has been implicated in quorum sensing signaling within the human host [24].

#### Intracellular signaling

Cyclic-di-GMP is an intracellular second messenger that integrates environmental signals and has been demonstrated to regulate several distinct cellular processes in vibrios such as motility, biofilm formation, virulence, and rugose colony morphology [25]–[27]. This signaling molecule has been proposed to be a critical component for the transition of *V. cholerae* from the aquatic environment to the host [28]. The intracellular concentrations of this second messenger are regulated by diguanylate cyclases (DGCs) and phosphodiesterases (PDEs) which synthesize and degrade c-di-GMP, respectively. Studies have shown that phosphodiesterase activity of the response regulator, VieA, reduces intracellular c-di-GMP levels which allows for optimal gene expression of virulence factors, specifically *toxT*, the transcriptional activator of toxin-coregulated pili (*tcp*) (the major colonization factor of *V. cholerae*) as well as cholera toxin (*ctx*) [28]. Furthermore, through the action of c-di-GMP, VieA negatively regulates expression of *vps* genes which contribute to exopolysaccharide production and biofilm formation in *V. cholerae* [29]. A similar regulatory response system (RocR/SadR) has been identified in *Pseudomonas aeruginosa* which was also shown to control biofilm and virulence phenotypes in a reciprocal manner in response to environmental signals [30]. Interestingly, our study found the sensor histidine kinase *vieS* and the cognate response regulator *vieA* to be significantly upregulated in *V. vulnificus* when exposed to human serum. Considering that *V. vulnificus* appears to lack *toxT*, *tcp* and *ctx* homologs, the downstream effect of this two-component system deserves further investigation as it may play a critical role in the transition from environment to host, as is the case for *V. cholerae*.

In *V. cholerae*, the ToxR regulon encodes over 20 genes that aid in intestinal colonization, toxin production and survival within the host [31]. Homologs of the *toxRS* operon have been identified in *V. vulnificus* and the ToxRS signal transduction system has been shown to stimulate hemolysin production and alter the outer membrane protein expression profile [32]. In addition to upregulation of *toxRS* expression in HS, we found increased expression of three hemolysins, along with differential expression of several outer membrane proteins.

Intracellular signaling also occurs through quorum sensing (QS) in which signaling molecules (autoinducers) accumulate extracellularly, bind a membrane-bound sensor to effect a collective change in gene expression patterns within the population. This results in the modulation of processes such as bioluminescence, biofilm formation, virulence, and fitness factor production (see [33] for a review on QS). Three systems of bacterial communication exist in Gram-negative bacteria and each system utilizes chemically different autoinducers. Only one functional quorum sensing pathway (autoinducer-2, involving interspecies communication) has been described in *V. vulnificus*, but it has been proposed that this bacterium possesses other, not yet identified, compensatory channels for quorum sensing [34]. The AI-3 system involves inter-kingdom cross-signaling in which bacteria can sense and respond to host-derived signals [35]. The sensor kinase, QseC, of this two-component signaling system acts as an “adrenergic receptor” that senses and responds to epinephrine, norepinephrine, and AI-3 in enterohemorrhagic *E. coli*, subsequently activating virulence genes [35], [36]. Interestingly, we identified the *qseBC* homolog in *V. vulnificus* to be upregulated in cells exposed to HS. Whether the QseBC system functions as a genuine inter-kingdom signaling network for *V. vulnificus* is an area of research that deserves prompt investigation as this system may be used to detect and respond to eukaryotic adrenergic signals present in the GI tract and/or human serum, thereby assisting the bacterium's navigation throughout the human host.

It is unclear whether *V. vulnificus* employs AI-1 (intraspecies) signaling as a LuxI/LuxR pair has yet to be genetically identified in this species [37], [38]. However, we identified a gene encoding a LuxR-family transcriptional regulator to be upregulated in human serum. In AI-1 signaling, LuxR serves as the response regulator which binds AHL signals and subsequently interacts with DNA to effect changes in gene expression in response to the stimulus. Interestingly, many bacteria (both with and without AI-1 signaling capabilities) have been found to possess LuxR-family proteins for which there is no obvious cognate LuxI synthase [39]. Recent research suggests these “LuxR-family orphans” may help in fine tuning of existing QS regulatory networks, control independent regulons, or allow bacteria to detect and respond to neighboring interspecies or inter-kingdom signals [39], [40]. Indeed, these propositions suggest the need to investigate the relevance of these LuxR-family orphans in pathogens such as *V. vulnificus*.

#### Response to heat/protein stabilization

Exposure to temperature stress can have deleterious effects on cellular components, particularly due to protein denaturation. In response, bacterial cells will activate the heat shock response (HSR) in order to protect the cell from physiological stress. This signaling pathway is governed by the transcription factor, σ^32^, which rapidly induces transcription of several heat-shock proteins (HSPs) including molecular chaperones and ATP-dependent proteases that aid in repair or disposal of damaged proteins. Exposing *V. vulnificus* to HS at 37°C (compared to ASW at room temperature) resulted in a remarkable upshift in σ^32^, hsp chaperones, and proteases. Interestingly, this effect was still prominent at two hours of incubation in serum. HSR typically occurs within minutes of temperature upshift and reaches a new steady-state level within ca. 30 m [41]. Considering the cross-protective qualities of HSPs [42], it is plausible that these genes also promote cellular stability in the presence of antimicrobial constituents within human serum.

#### Toxins and exoenzymes

*V. vulnificus* produces cytotoxic effects to human cells by secreting hemolysins and extracellular proteases [3]. Hemolysins, such as the *vvhA* cytolysin, cause cellular damage by forming pores in the cell's membrane which can lead to apoptosis, vascular permeability and hypotension [43]. Extracellular proteases, such as the metalloprotease *vvpE* and elastase, cause tissue necrosis and cutaneous lesions as well as vascular permeability and edema. While the pathogenic effects of these putative virulence factors have been thoroughly investigated, some studies indicate that they may not play a significant role in virulence [44]–[46]. This conclusion is supported in our current study, which found no change in expression of *vvhA* between ASW and HS, and down-regulation of *vvpE* and elastase in HS. However, we found three other putative hemolysins and several proteases (including two zinc-dependent proteases and two collagenases) to be upregulated in human serum. Further evaluation of these putative toxins is necessary to assess if and how they contribute to virulence.

### Genes induced in artificial seawater

#### Transcription factors

In *V. vulnificus*, the alternative sigma factor, RpoS, aids in adaptation to environmental stress (such as osmotic shock, nutrient stress, and oxidative stress) and has been shown to be required for full motility [47]. We found *rpoS* expression to be enhanced in ASW, highlighting the necessity of the general stress response in the environment. Additionally, we found the Crl transcriptional regulator to be upregulated in ASW. In *E. coli*, Crl is an auxiliary factor that binds to σ^S^ and enhances its affinity for certain promoters, including the genes necessary for curli fimbriae formation [48]. These extracellular matrix components facilitate surface colonization and biofilm formation, and expression is positively regulated by curli subunit gene D (CsgD) [48], [49]. Previous studies have identified CsgD homologs in *V. vulnificus, V. parahaemolyticus*, and *V. cholerae* [50]. In *V. cholerae*, the CsgD homolog, VpsT, has been shown to enhance biofilm formation and activate genes involved in VPS exopolysaccharide production [51]. In our study, expression of a VpsT homolog was upregulated in *V. vulnificus* cells (CMCP6 only) in ASW.

Expression of the gene encoding the alternative sigma factor RpoN (σ^54^) was also upregulated in ASW. This transcription factor regulates over 30 operons in *E. coli*, nearly half of which are involved in nitrogen assimilation and metabolism [52]. The remaining σ^54^-dependent genes have been proposed to neutralize adverse conditions and/or prevent depletion of metabolites and energy resources in limiting environments [52]. In some *Vibrio* spp., σ^54^ has been documented to upregulate genes involved flagellar synthesis and motility [53]–[55]. Interestingly, deletion of *rpoN* in *V. cholerae* reduces the competitive fitness of cells (relative to the wild-type) when colonizing the infant mouse model, however this deletion enhances fitness in intestinal colonization by *V. parahaemolyticus* in the adult mouse model [53], [55]. Furthermore, this sigma factor has been implicated in regulating EPS in *V. vulnificus* and *V. fischeri* [56], [57]. Thus, in ASW, RpoN may be regulating a number of processes including nitrogen metabolism, motility, colonization, and biofilm formation and further investigation into this regulatory network in *V. vulnificus* is prompted.

IscR is a transcription factor that regulates the *isc* operon in *E. coli* and controls the expression of over 40 genes including those involved in Fe-S cluster biosynthesis, anaerobic respiration, and biofilm formation [58]. Recently, an IscR homolog was identified in *V. vulnificus* that regulates a number of genes possibly involved in pathogenesis, including genes involved in motility and adhesion, hemolytic activity, and oxidative stress response. Interestingly, our study revealed upregulation of five out of eight genes within the *isc* operon, including *iscR*, when cells were exposed to ASW. Additionally, IscR regulated genes involved in chemotaxis, methyl-accepting chemotaxis proteins, and a glutaredoxin were also upregulated in our study, reaffirming the relationship identified by Lim et al. (2014). While IscR appears to play a critical role in protecting the cell from reactive oxygen species generated by host cells during infection [59], it may be that IscR also plays an important role in environmental survival.

#### Adherence and Biofilm formation

The *tad* (tight adherence) genes encode the machinery needed to assemble Flp pili (fibrils) that mediate adherence to surfaces. The *tad* genes have been found to be essential for adherence, biofilm formation, colonization, and pathogenesis in a number of genera, and are considered to be instrumental in the colonization of diverse environmental niches [60]–[63]. These genes are present on a genomic island referred to as the “widespread colonization island” which is present in a wide variety of bacteria, including several human pathogens [61]. Some species harbor more than one distinct tad locus, including *V. vulnificus*, which contains two loci on chromosome I and a third locus on chromosome II. One of these three *tad* loci was differentially expressed in our study with expression being enhanced in ASW relative to HS. The function(s) of the *tad* loci in *V. vulnificus* have yet to be investigated but it is reasonable to conjecture that this widespread colonization island provides important functions for this organism specifically regarding adherence and colonization. Indeed, a previous RNAseq study found that (when compared to growth in a nutrient-rich medium), a *V. vulnificus* biotype 3 strain isolated from a wound infection expressed the Flp-coding region [64].

Additionally, we found several genes involved in biogenesis of the type IV mannose-sensitive haemagglutinin (MSHA) pilus to be upregulated in ASW. In V. cholerae, MSHA facilitates attachment to chitinous substrates, such as zooplankton exoskeletons, allowing for subsequent biofilm formation [65], [66]. In contrast to the toxin-coregulated pilus (a predominant virulence factor in *V. cholerae*), MSHA biogenesis has been shown to be repressed *in vivo*, acting as an “anticolonization factor” in the human host [67]. Our results suggest that MSHA may play a similar role in *V*. *vulnificus* in which this pilus mediates attachment to substrates in the aquatic environment.

*V. vulnificus* produces extracellular surface polysaccharides (EPS) and capsular polysaccharides (CPS) both of which are involved in biofilm formation. CPS is recognized as an essential virulence factor for *V. vulnificus* and negatively impacts biofilm formation [68], [69], whereas EPS production is essential for bacterial attachment and biofilm formation [57]. *V. vulnificus* has at least three kinds of EPS and we found one of these polysaccharide biosynthetic loci to be significantly upregulated in ASW. This set of EPS genes (homologous to the “*syp* genes” originally identified in *V. fischeri*) appears to be transcriptionally regulated by σ^54^ [56], and produces wrinkled colonies, pellicle formation, and matrix production when induced [70]. Upregulation of *syp*-like genes in ASW suggests this EPS locus may play an important role in environmental persistence.

#### Intracellular signaling and chemotaxis

As previously mentioned, the signaling pathway regulator c-di-GMP controls phenotypes associated with biofilm formation and planktonic growth in a reciprocal manner. Intracellular levels of c-di-GMP increase through the action of diguanylate cyclases (DGCs) which synthesize this second messenger. Subsequently, c-di-GMP levels can be reduced through the action of phosphodiesterases (PDEs) which degrade this molecule. We identified 17 DGCs and six PDEs to be upregulated in ASW, as well as two PDEs to be downregulated in ASW. This result suggests that cells in this environment have higher intracellular levels of c-di-GMP. In *V. vulnificus*, c-di-GMP positively regulates biofilm formation by regulating production of EPS, thus cells in ASW would presumably exhibit enhanced biofilm formation and possibly decreased virulence potential [25], [71]. This speculation is further supported through the observed upregulation of *rpoS* expression in ASW, as RpoS has been shown to modulate c-di-GMP levels by enhancing expression of DGCs.

Chemotaxis is initiated by membrane-bound chemoreceptors called methyl-accepting chemotaxis proteins (MCPs) which bind a ligand resulting in a signal transduction cascade to direct the flagellum's activity. The role of chemotaxis in the process of infection has been examined in several bacteria/enteric pathogens and for many of these, motility is an important virulence factor. Interestingly, chemotaxis in *V. cholerae* inhibits its ability to colonize the small intestine of infant mice and is inversely regulated with expression of virulence traits [72].

*V. cholerae* has three chemotaxis gene clusters, denoted *cheA*-1, *cheA*-2, and *cheA*-3, although only *cheA*-2 has been found to be essential for chemotaxis in growth media [73]. In the current study, we identified two of these homologs (*cheA*-2 and *cheA*-3) in *V. vulnificus* and found the *cheA*-3 homolog (located on chromosome II) to be upregulated in ASW. Additionally, many of the MCPs located throughout the genome were significantly expressed in this condition. In *V. cholerae cheA*-3 is not required for chemotactic control of flagellar motility, thus it has been suggested that this operon could regulate flagellum-independent motility (e.g. twitching motility) [73], a proposition that could be extended to *V. vulnificus*. The intimate relationship between chemotaxis and virulence expression that has been documented in *V. cholerae* prompts further investigation into these mechanisms in *V. vulnificus*. For both of these pathogens, chemotaxis is assuredly a vital fitness factor in both environmental and pathogenic settings.

*V. vulnificus* C-genotypes possess an operon homologous to the RsbRST stress module (or stressosome) which is classically found in Gram-positive bacteria such as *Bacillus subtilis* and *Staphylococcus aureus* [11] and governs the “general stress response”, sometimes playing a role in virulence. This supramolecular signaling complex perceives signals related to environmental stress or nutrient limitation, triggering a signal transduction phospho-relay that ultimately activates the sigma factor (σ^B^), which will then bind to the core RNA polymerase to direct transcription of over 150 genes involved in stress adaptation [74]. For organisms such as *V. vulnificus* that possess stressosome orthologs, but lack σ^B^, the stressosome has been proposed to be involved in regulating aerotaxis, two-component signaling systems, and the biosynthesis of secondary messenger signaling molecules [74]. We assessed 20 C-genotypes and 29 E-genotypes for the presence of this operon and found this module to be specific to C-genotypes, with 75% of C-genotypes and none of E-genotypes possessing the entire operon (**S1 Table**). Furthermore, we found these genes to be upregulated in ASW relative to HS, suggesting that the stressosome serves an important function for the organism.

**Figure 3:**
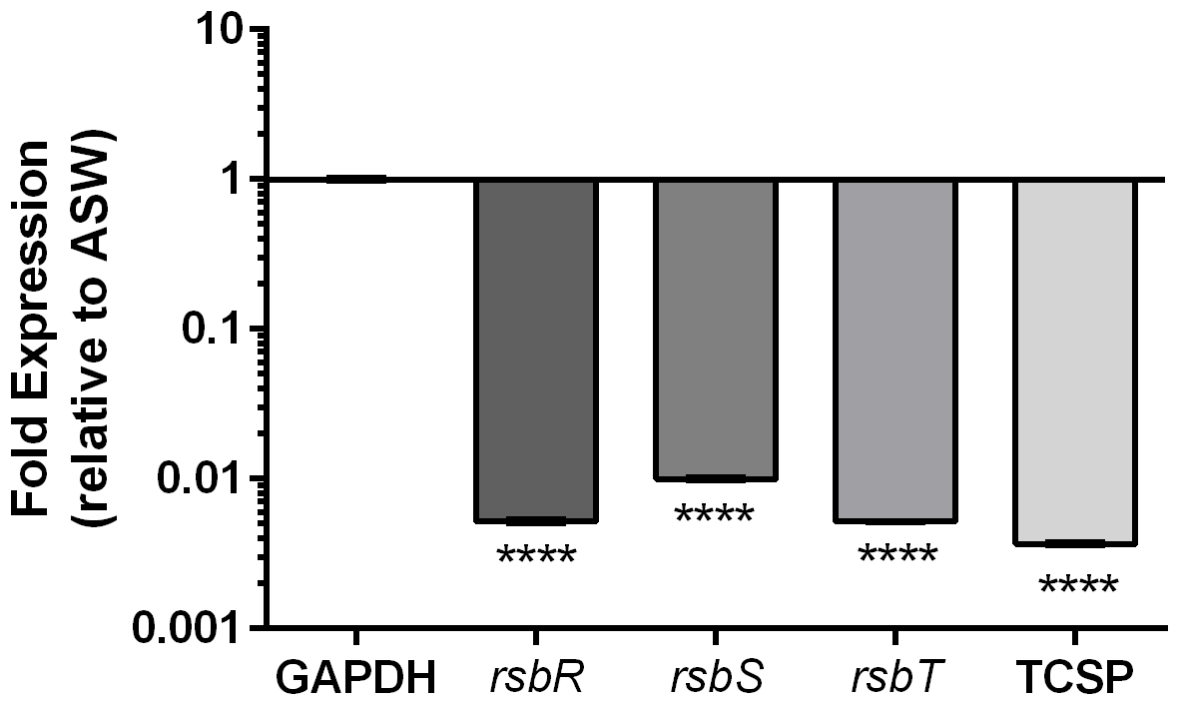
Gene expression of the stressosome in human serum relative to artificial seawater. Relative qRT-PCR was performed on CMCP6 to examine expression of select stressosome genes (*rsbRST* and a downstream two-component sensory protein, here denoted “TCSP”) confirming RNAseq results. GAPDH was used to normalize expression data. Asterisks represent significant differences in expression p<0.0001 (One-way ANOVA).

**Figure 4:**
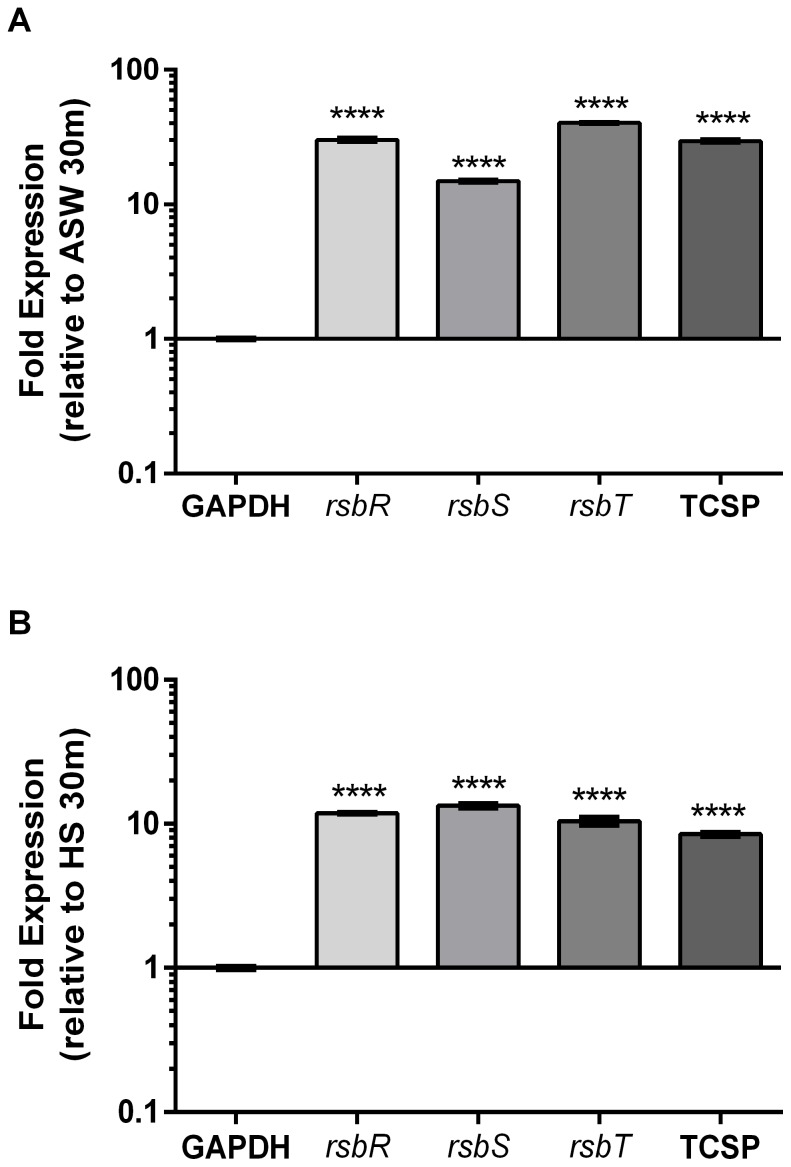
Temporal gene expression of the stressosome during exposure to A) artificial seawater or B) human serum. Relative qRT-PCR was performed on CMCP6 to examine expression of select stressosome genes (*rsbRST* and a downstream two-component sensory protein, here denoted “TCSP”) after incubation in each condition for 2 hrs relative to 30 min. GAPDH was used to normalize expression data. Asterisks represent significant differences in expression p<0.0001 (One-way ANOVA).

**Figure 5:**
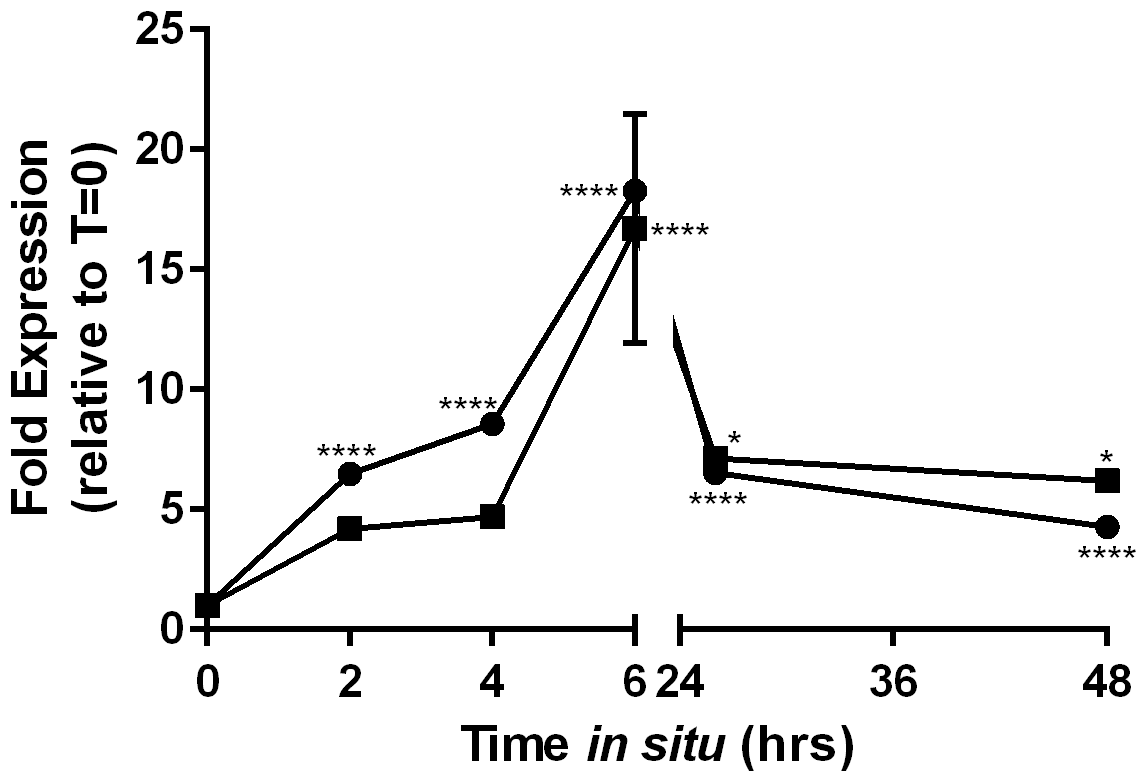
Temporal gene expression of the stressosome in natural estuarine waters. Cells of CMCP6 were incubated in natural estuarine waters for 2 days using membrane diffusion chambers. Relative qRT-PCR was performed on samples collected at several time points and expression of select stressosome genes (*rsbR*, circles; *rsbT*, squares) was analyzed relative to 0 hrs of incubation (prior to *in situ* exposure). GAPDH was used to normalize expression data. Asterisks represent significant differences in expression (One-way ANOVA).

We examined gene expression of the *rsbRST* operon using relative qRT-PCR and found these genes to be repressed more than 100-fold in HS relative to ASW (**Fig. 3**), validating our RNAseq transcriptome findings. To further characterize expression of the stressosome we examined how this operon is regulated temporally using relative qRT-PCR. Interestingly we found stressosome genes to be upregulated over time in ASW (**Fig. 4A**), as well as in HS (**Fig. 4B**), albeit to a lesser extent. The enhanced expression of stressosome-related genes in ASW relative to HS suggests an ecological function for this system, thus we used membrane diffusion chambers to examine temporal gene expression of *rsbR* and *rsbT*, *in situ*. When placed in their native estuarine environment, cells upregulated *rsbR* and *rsbT* over 10-fold within 6 hours and continued to express these genes for up to 48 hrs (**Fig. 5**).

Further studies are warranted to assess if and how the stressosome module contributes to pathogenicity and/or niche colonization in strains of *V. vulnificus* harboring these genes. It may be that this system has been optimized to allow for integration of various signals into a single output, thereby allowing for fine-tuning of stress responses in a particular environment. Expression of this operon *in situ* also suggests a role for this system in the environment and it may have been acquired to aid in niche expansion.

## Conclusions

**Figure 6:**
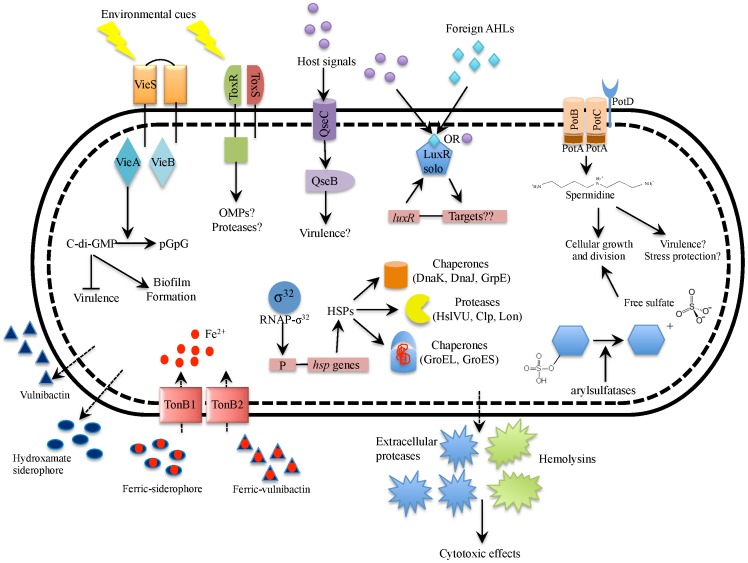
Diagrammatic summary of genes upregulated in human serum relative to artificial seawater. Depicted in this figure are genes (or sets of genes) upregulated in HS along with the inferred phenotypic outcomes. In this condition, the cell scavenges for nutrients by utilizing processes involved in substrate binding and transport including iron chelators and transport systems (hydroxamate-siderophore, vulnibactin, TonB1, and TonB2), spermidine transport (PotABCD), and sulfate scavenging arylsulfatases. In response to unknown environmental cues, the cell also engages in intracellular signaling using systems such as a two component signal transduction system (VieSA) involved in c-di-GMP signaling, the ToxRS signal transduction system, and a LuxR-family orphan possibly involved in quorum sensing signaling, and the cell possibly senses and responds to host hormones using a two-component signaling system (QseBC) involved in inter-kingdom cross-talk. The cell responds to human body temperature (37°C) by upregulating genes involved in the heat shock response (sigma factor RpoH, chaperones and proteases). The cell also secretes several putative virulence factors such as cytotoxic hemolysins and extracellular proteases. Note, this diagram is developed based solely on transcriptomic data and further investigation is necessary to establish the proposed model.

The goal of the current study was to investigate the transcriptome profile of two clinically isolated C-genotypes of the human pathogen, *V. vulnificus*. Overall, this study unveiled a picture of the “virulence profile” cells exhibit in human serum and the “environmental profile” displayed in cells exposed to estuarine-like conditions. From this picture emerges a phenotypic dichotomy that closely resonates with previous findings regarding the dual life-style of *V. cholerae*. **Figs. 6** **and** **7** feature diagrams of differentially expressed genes that were upregulated in human serum and artificial seawater and attempt to depict the potential phenotypic outcomes for existence in these two diverse environments. As with all high-throughput methods, a detailed investigation of the current results is necessary to establish their functional relevance for bacterial regulation, physiology and pathogenicity.

**Figure 7:**
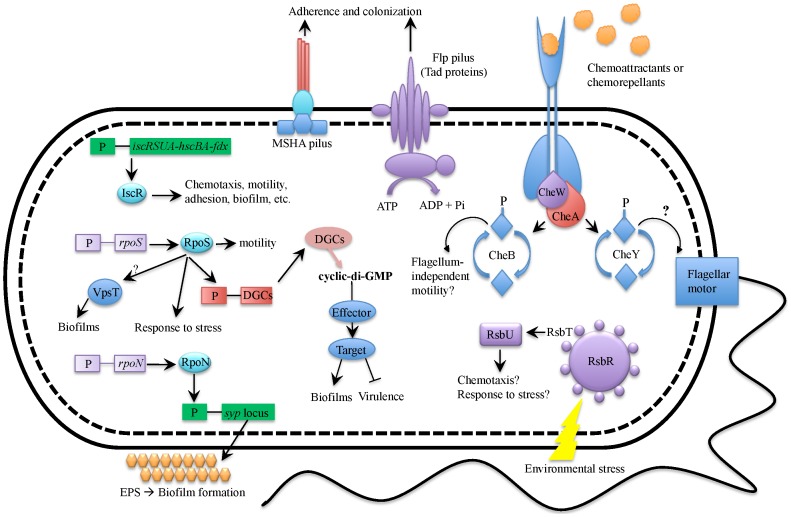
Diagrammatic summary of genes upregulated in artificial seawater relative to human serum. Depicted in this figure are genes (or sets of genes) upregulated in ASW along with the inferred phenotypic outcomes. The cell expresses several transcription factors involved in stress response, motility, and biofilm formation (RpoS, RpoN and IscR). The cell also expresses genes involved in attachment and biofilm formation including Flp pilus (tad locus), MSHA pilus, and EPS (syp locus). Through the action of diguanylate cyclases (DGCs), the cell has high intracellular concentrations of c-di-GMP which enhances biofilm formation and possibly reduces virulence factor production. In response to chemoattractants and chemorepellents, the cell also engages in chemotactic sensing and signaling which may result in modulation of flagellar rotation and/or flagellum-independent motility. As a result of some unknown environmental cue, the cell also expresses genes involved in the stressosome module (RsbRSTU) which is likely involved in chemotaxis and/or stress response. Note, this diagram is developed based solely on transcriptomic data and further investigation is necessary to establish the proposed model.

Our study reveals that when *V. vulnificus* cells encounter human serum, they exhibit a “virulence profile” similar to what has been seen in the initial stage of *V. cholerae* infection [28], [31], in which a number of genes encoding sensory proteins (*vieSA*, *toxRS*) and virulence-associated genes (such as *toxRS*, *vieSA*, hemolysins and proteases) and were upregulated in this environment (Fig. 6). Additionally, expression of the *qseBC* operon (a system which has been previously shown to be involved in inter-kingdom communication in other organisms) was also upregulated in HS. Regulation of this system is a finding which we believe to be novel for *V. vulnificus*.

In artificial seawater, genes involved in stress response, chemotaxis, adhesion, and biofilm formation were upregulated (Fig. 7). In *V. cholerae*, expression of many chemotaxis genes and motility genes are dependent on RpoS, which has been shown to orchestrate the “mucosal escape response” in the later stages of infection, and coincides with down-regulation of virulence-gene expression [75]. This switch in genetic programming is thought to prepare the organism for the next stage in its life cycle, dissemination back into the environment. Here, we discovered a similar genetic profile in which transcription factors including *rpoS*, and genes involved biofilm formation (e.g. EPS and pili-associated genes) were enhanced, and this phenotype appeared to be governed (at least in part) through c-di-GMP signaling. We also observed upregulation of a distinct signaling network, referred to as the stressosome in distantly related bacteria, which is unique to C-genotypes of *V. vulnificus.* This is the first report to investigate expression of this enigmatic network in *V. vulnificus*, and we believe future investigations will be fruitful in enhancing our understanding of this human pathogen.

To our knowledge, this is the first transcriptome-based analysis of *V. vulnificus* biotype 1 strains, and we identified a number of genes which appeared to “trend” together. These findings provide a more holistic vision of how *V. vulnificus* behaves in a given environment, and provides a foundation on which future studies on this human pathogen can be built. Currently, we are in the process of investigating the transcriptome response of E-genotype cells of *V. vulnificus* in the same conditions in order to compare 1) how this genotype responds to the transition from environment to host, and 2) how C- and E-genotypes differentially express genes within the same environment.

## Materials and Methods

### Experimental design and sample preparation

#### Experimental design

Two clinically isolated strains of *V. vulnificus* (CMCP6 and YJ016) were selected due to availability of their genome sequences. Strains were grown in heart infusion (HI) broth (BD, New Jersey) at 30°C overnight at 200 rpm and then diluted 1∶100 v:v into fresh HI and grown at 30°C for ca. 90 m at 200 rpm until cells reached logarithmic phase (OD_610_ 0.15–0.25). Cells were pelleted at 5000×g at room temperature for 10 m, resuspended in 10 parts per thousand (ppt) artificial seawater (ASW), and inoculated into either 10 ppt ASW or normal human serum (MP Biomedicals) to a final concentration of ca. 1e8 CFU/ml. Cells were incubated in 10 ppt ASW at room temperature (22°C), or human serum at 37°C, on a rotisserie for 2 hours. ASW at 22°C was chosen to represent a controlled version of the natural estuarine waters and temperature this bacterium encounters in its native environment. Human serum at 37°C was chosen to reflect the host environment and human body temperature. Pooled human serum (HS) used in this study was collected from male donors and contained nutrients in the form of glucose (96 mg/dl) with total protein levels of 5.5–7.5 g/dl [76].

#### RNA extraction and purification

Two biological replicates were performed for each condition and after 2 hours of incubation cells were treated with 2 volumes of RNAprotect (Qiagen) following manufacturer's instructions. Preserved pellets were resuspended in TE buffer, pH 8.0 (Ambion) with 1 mg/ml lysozyme (Sigma Aldrich) and vortexed at medium speed for 30 m. RNA was extracted using RNeasy Midi Kit (Qiagen) following protocol with on-column DNase I treatment. RNA was eluted from the column using nuclease-free water and a second post-extraction DNase treatment using TURBO DNA-free (Life Technologies) following the rigorous DNase treatment protocol. RNA quality and quantity was assessed using a NanoDrop spectrophotometer (Thermo Scientific) and RNA samples having a 260/280 ratio <1.7 were not used in downstream applications.

To confirm the complete removal of DNA, endpoint PCR was performed on RNA samples using Promega's Go-Taq DNA polymerase, 5X Green GoTaq Reaction Buffer, 10 mM dNTP mix, and primers targeting the species-specific *vvhA* gene [7]. Cycling parameters were according to the manufacturer's recommendations, with an annealing temperature of 53.1°C and 40 cycles of amplification. Any amplification of the *vvhA* gene was indicative of DNA contamination in which case the RNA was not used for downstream processes.

RNA integrity was assessed using an Agilent 2100 Bioanalyzer and 6000 Nano kit (Agilent Technologies). The 23S/16S rRNA ratio and RNA integrity number (RIN) were confirmed to be >1.8 and close to 10, respectively. Due to the enhanced sensitivity of this technique, the electropherogram for each sample was also screened for residual DNA contamination. Samples yielding bands >4000 nt were not used in downstream applications. Total RNA samples were quantified using a NanoDrop Spectrophotometer (Thermo Scientific).

#### mRNA Enrichment and cDNA Library Preparation

To enhance the sequencing coverage of mRNA transcripts, we depleted highly expressed rRNA transcripts using Epicentre's Ribo-Zero kit (Epicentre Biotechnologies). Remaining mRNA was assessed for quality and levels of rRNA contamination using the Agilent Bioanalyzer and RNA 6000 Pico Chip (Agilent Technologies). Samples having >1% rRNA contamination were not used for downstream applications. mRNA was quantified using Qubit's 2.0 Fluorometer and RNA High Sensitivity Assay Kit (Life Technologies).

DNA libraries complementary to mRNA sequences were prepared for each sample using ScriptSeq v2 RNA-Seq Library Preparation Kit (Epicentre Biotechnologies). This kit, compatible with Illumina Technologies, enabled directional, paired-end sequencing. Libraries were prepared following manufacturer's instructions. The size and quality of each cDNA library was assessed using Agilent Bioanalyzer's High Sensitivity DNA kit (Agilent Technologies). Insert sizes were approximately 400 bp+/−35 bp. cDNA library quantification was performed using Qubit's 2.0 Fluorometer and Quant-iT dsDNA High Sensitivity Assay Kit (Life Technologies). Samples were stored at −20°C and transported to the David H. Murdock Research Institute (Kannapolis, N.C.) for sequencing using Illumina's Genome Analyzer.

### RNA sequencing data analysis and statistics

#### Data preprocessing

Approximately 61 to 79 million paired-end raw reads were generated for each set of biological replicates (**S2 Table**). RNA sequencing data is publicly available in NCBI's BioProject database (BioProject ID PRJNA252365). Raw Illumina sequence reads were filtered to remove reads which fell below the sequencer quality threshold. A second round of filtering using fastq-mcf [77] removed low-quality regions of sequence using a quality trimming window of size 4 and a minimum quality score of 15. The resulting reads were of high average quality and read lengths were tightly clustered around 100 nucleotides. The remaining filtered reads were aligned to reference genomes using Bowtie2 [78]. The alignment rate using Bowtie2 with default settings for paired-end reads was greater than 95% for all samples in the study indicating high quality and reproducibility of the data set (**S2 Table**). The view and sort functions of SAMTools [79] were used to convert the aligned read data into the correct format for subsequent analysis.

#### Expression analysis

Read counts were summarized against the CMCP6 and YJ016 reference genomes using FeatureCounts [80]. Differential gene expression analysis was performed using the EdgeR package [81] following the approach outlined by Jenkins [82]. EdgeR identifies significant differentially expressed genes out of genome-scale count data using exact tests based on the negative binomial distribution. Replicate data for each condition was used for normalization using the TMM method [83]. The artificial sea water (ASW) condition was treated as the baseline condition for analysis. Genes identified as differentially expressed in human serum (HS) relative to ASW, with a p-value less than 0.0001 were selected for further analysis.

#### Gene Ontology (GO) enrichment analysis

Gene category enrichment analysis was performed using the Ontologizer software [84]. The study sets of differentially expressed genes generated by EdgeR were compared to the population set of GO-annotated genes in the published CMCP6 and YJ016, respectively. The Ontologizer makes several different enrichment analysis methods available. The Parent-Child Union method, which avoids detection artifacts due to the hierarchical structure of the GO, was used to identify enriched GO term categories, and the Benjamini-Hochberg [85] method for multiple-testing correction was applied in order to minimize the false discovery rate. Reference Gene Ontology OBO file [86] and genome-specific GO annotations for *V. vulnificus* CMCP6 and YJ016 were downloaded from UniProt-GOA [87] on April 1, 2014. 3066 genes in CMCP6 and 3107 genes in YJ016 had available GO term mappings, resulting in 331 of 469 DE genes in CMCP6 and 406 of 661 DE genes in YJ016 being included in the enrichment analysis. Differential expression is summarized by GO terms identified as enriched in the study set relative to the population set, with a p-value <0.05.

### PCR and qRT-PCR analysis of stressosome module

#### PCR analysis of RsbRST stressosome module

Using PCR, 20 *V. vulnificus* C-genotypes and 29 E-genotypes were assessed for the presence of stressosome-associated genes (*rsbR*, *rsbS*, *rsbT*, *rsbU*, and a downstream two-component sensor protein). Primers (Sigma Aldrich) were designed for each gene using all three sequenced C-genotype strains of *V. vulnificus* (CMCP6, YJ016, and M06-24) reported in NCBI's database. Primer sequences are listed in **S3 Table**. Optimal primer quality and fidelity were assessed using IDT OligoAnalyzer 3.1 software, and primer specificity was initially analyzed using *in silico* PCR [88]. DNA extracted from each strain was subjected to 30 cycles of PCR with Promega's 5X Green Go*Taq* Reaction Buffer, 10 mM PCR Nucleotide Mix, 1.25 U Go*Taq* DNA Polymerase, and 0.5 µM of each primer. Thermal cycling parameters were followed according to manufacturer's recommendations (Promega) and PCR products were visualized by gel electrophoresis on 1% agarose gels stained with ethidium bromide.

#### Gene expression of RsbRST stressosome module

Gene expression of selected genes associated with the stressosome module was examined in human serum relative to 10 ppt ASW using relative quantitative reverse transcription-PCR (qRT-PCR) as previously described [89]. Primers were designed to be suitable for qRT-PCR and PCR amplification efficiency was assessed using parameters previously described [89]. Cells of CMCP6 were treated and RNA was extracted as described in the “experimental design and sample preparation” section above. Total RNA (1 µg) was reverse transcribed using qScript cDNASuperMix (Quanta Biosciences) and 50 ng of cDNA template was carried over for quantitative PCR (qPCR). qRT-PCR was performed on three technical replicates for each sample using PerfeCTa SYBR green FastMix, LowROX (Quanta Biosciences). Negative controls and “no-RT” controls were employed to rule out the influence of DNA contaminants or residual genomic DNA, respectively. Expression levels of each gene were normalized by using an endogenous control gene (glyceraldehyde-3-phosphate dehydrogenase [GAPDH]) to correct for sampling errors. Fold changes in expression levels were measured using the Pfaffl equation [90], taking into account the differences in PCR efficiencies between primer sets. Significant differences in gene expression were assessed using a one-way analysis of variance (ANOVA) followed by Bonferroni's post hoc test for multiple comparisons. Significance was determined by using a 95% confidence interval. Gene expression data were analyzed by using GraphPad Prism (version 5.0; GraphPad Software Inc.).

To further investigate the expression pattern of the stressosome module, gene expression was analyzed over time in human serum and 10 ppt ASW (2 hrs relative to 30 min) using the protocol described above. Additionally, *in situ* gene expression by cells incubated in natural estuarine waters was also performed as previously described [91]. Briefly, laboratory grown cells were diluted into 15 ppt ASW, injected into sterile (0.22 µm) membrane diffusion chambers, and deployed in estuarine waters along Core Creek in Beaufort, North Carolina. Chambers were deployed in September of 2012 and samples were collected periodically for up to 2 days. For the duration of the experiment, estuarine water temperatures ranged from 13.8°C to 17.2°C and salinity ranged from 17 ppt to 22 ppt. Samples were treated with RNAprotect (Qiagen) within 10 minutes of collection. RNA extraction followed by qRT-PCR was performed as previously mentioned.

## Supporting Information

S1 Figure Smear plot of differentially expressed genes in *V. vulnificus* YJ016 exposed to human serum (relative to artificial seawater). The smear plot shows the relationship between the log fold change and log counts per million. Green points represent non-significant DE genes whereas red points show genes that are significantly differentially expressed (p<0.0001) in relation to artificial seawater.
(TIFF)

S2 Figure Linear compressed view of differentially expressed genes in *V. vulnificus* exposed to human serum (relative to artificial seawater). These charts show a compressed view of the differentially expressed genes in *V. vulnificus* YJ016 by chromosome (top; chromosome I, bottom; chromosome II) and allows quick identification of clusters of differentially expressed genes, both positive and negative. The y-axis shows the log fold change and the x-axis is the nucleotide position of the chromosome. Blue bars represent non-significant DE genes whereas red bars show genes that are significantly differentially expressed (p<0.0001) in relation to artificial seawater.
(TIFF)

S1 Table Presence or absence of stressosome genes in *V. vulnificus* strains by PCR analysis.
(DOCX)

S2 Table Summary of RNAseq coverage data using the Illumina genome analyzer.
(DOCX)

S3 Table Primers designed for this study.
(DOCX)

S1 File Significantly differentially expressed gene lists for CMCP6 and YJ016 (p<0.0001).
(XLSX)

S2 File Select differentially expressed genes upregulated in human serum.
(XLSX)

S3 File Select differentially expressed genes upregulated in artificial seawater.
(XLSX)
