# Supplementary figures and images for: Transcriptome Sequencing Reveals the Virulence and Environmental Genetic Programs of Vibrio vulnificus Exposed to Host and Estuarine Conditions
Source: PLoS One. 2014 Dec 9;9(12):e114376. doi: 10.1371/journal.pone.0114376 (PMC4260858; doi:10.1371/journal.pone.0114376)

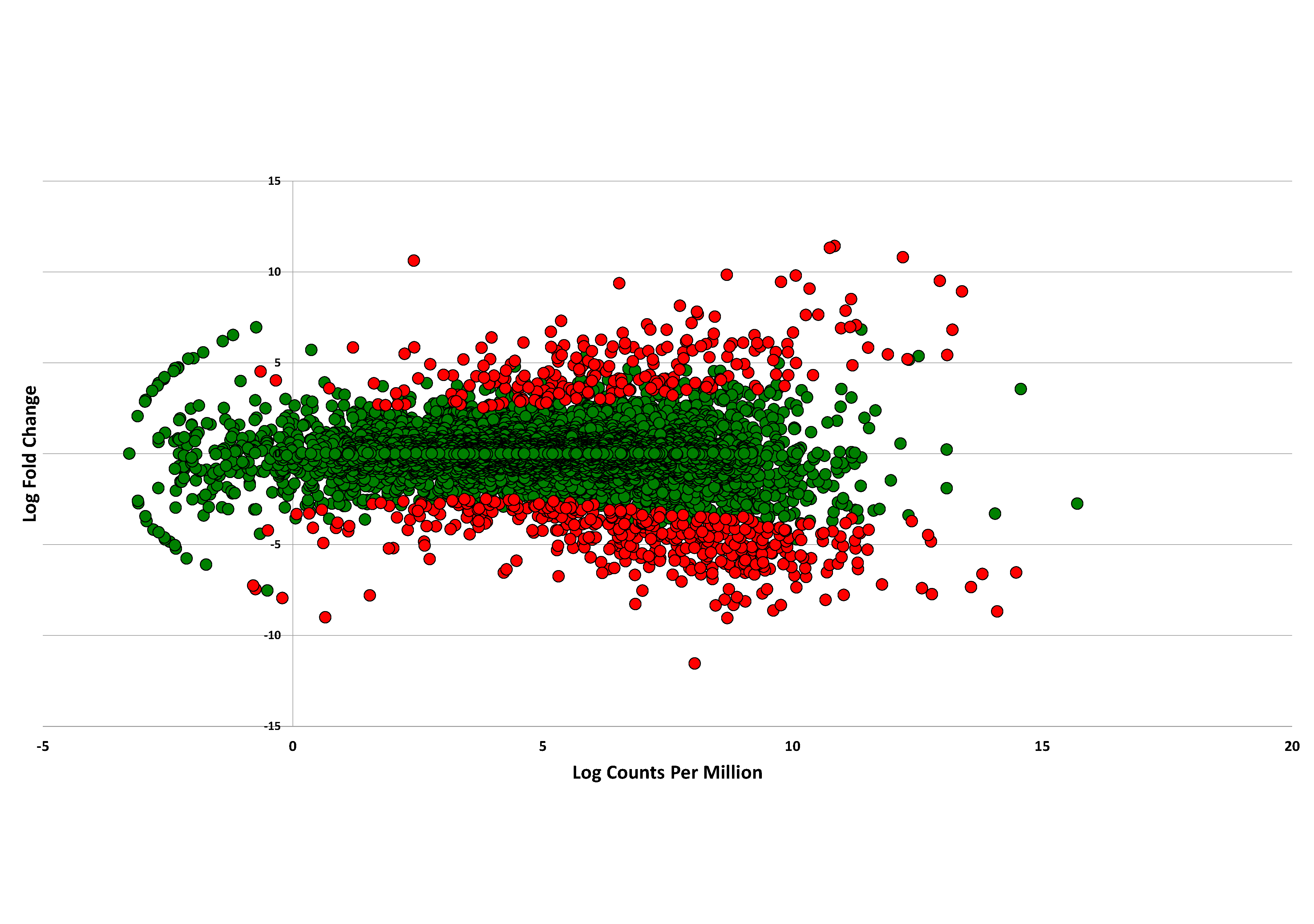

Supplement: S1 Figure — Smear plot of differentially expressed genes in V. vulnificus YJ016 exposed to human serum (relative to artificial seawater). The smear plot shows the relationship between the log fold change and log counts per million. Green points represent non-significant DE genes whereas red points show genes that are significantly differentially expressed (p<0.0001) in relation to artificial seawater. (TIFF) [file pone.0114376.s001.tiff]

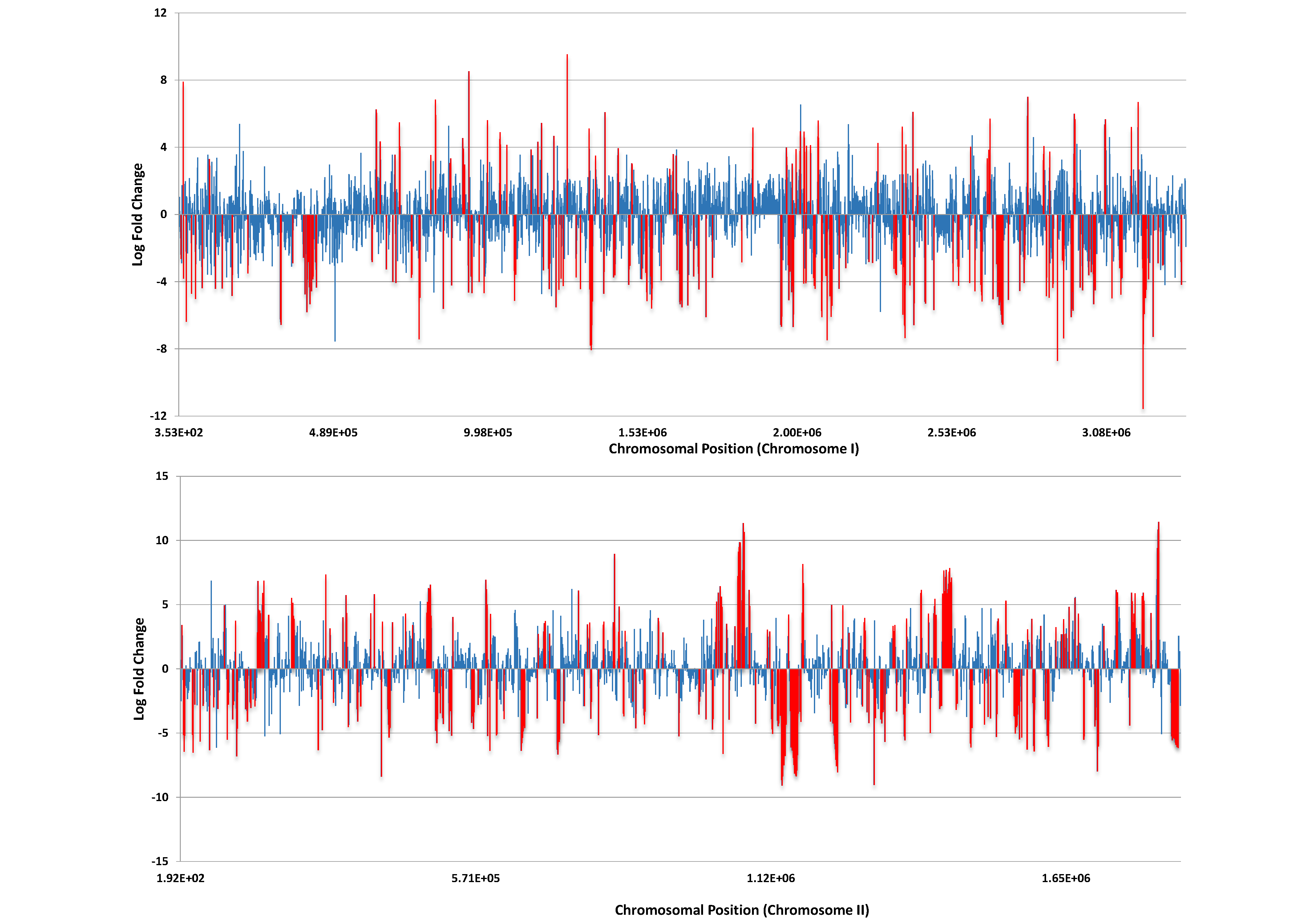

Supplement: S2 Figure — Linear compressed view of differentially expressed genes in V. vulnificus exposed to human serum (relative to artificial seawater). These charts show a compressed view of the differentially expressed genes in V. vulnificus YJ016 by chromosome (top; chromosome I, bottom; chromosome II) and allows quick identification of clusters of differentially expressed genes, both positive and negative. The y-axis shows the log fold change and the x-axis is the nucleotide position of the chromosome. Blue bars represent non-significant DE genes whereas red bars show genes that are significantly differentially expressed (p<0.0001) in relation to artificial seawater. (TIFF) [file pone.0114376.s002.tiff]
