# Supplementary material for: Transcriptome Sequencing Reveals the Virulence and Environmental Genetic Programs of Vibrio vulnificus Exposed to Host and Estuarine Conditions
Source: PLoS One. 2014 Dec 9;9(12):e114376. doi: 10.1371/journal.pone.0114376 (PMC4260858; doi:10.1371/journal.pone.0114376)
Supplement: S1 Table — Presence or absence of stressosome genes in V. vulnificus strains by PCR analysis. (DOCX) [file pone.0114376.s003.docx]

**Table S1. Presence or absence of stressosome genes in *V. vulnificus* strains by PCR analysis.**

| **Strain Name** | **Genotype** | **Presence of Stressosome module** |
| --- | --- | --- |
| CMCP6 | C | + |
| YJ016 | C | + |
| M06-24 | C | + |
| C7184 | C | + |
| UNCC 1002 | C | - |
| UNCC 913 | C | - |
| EDL -174 | C | + |
| SPRC 10143 | C | + |
| LSU 1866 | C | + |
| L-180 | C | + |
| A48 | C | - |
| LSU 1003 | C | - |
| LSU 1007 | C | + |
| LSU 1009 | C | + |
| LSU 1015 | C | + |
| LSU 1365 | C | + |
| LSU 1456 | C | + |
| SPRC 10141 | C | - |
| SPRC 10217 | C | + |
| SPRC 10145 | C | + |
| LSU 1014 | E | - |
| VVL1 | E | - |
| BC 478 | E | - |
| LSU 2098 | E | - |
| LSU 549 | E | - |
| 93A-3097 | E | - |
| B9629 | E | - |
| SPRC 10113 | E | - |
| SPRC 10111 | E | - |
| A1402 | E | - |
| 9598-84 | E | - |
| SREL 97 | E | - |
| SREL 119 | E | - |
| MLT 192 | E | - |
| MLT 201 | E | - |
| SM1 | E | - |
| SM2 | E | - |
| SM3 | E | - |
| SM4 | E | - |
| SM5 | E | - |
| SM6 | E | - |
| SM7 | E | - |
| SM8 | E | - |
| A25 | E | - |
| JY1701 | E | - |
| JY1305 | E | - |
| ENV1 | E | - |
| SS108-A3A | E | - |
| E64MW | E | - |
