# Supplementary material for: Transcriptome Sequencing Reveals the Virulence and Environmental Genetic Programs of Vibrio vulnificus Exposed to Host and Estuarine Conditions
Source: PLoS One. 2014 Dec 9;9(12):e114376. doi: 10.1371/journal.pone.0114376 (PMC4260858; doi:10.1371/journal.pone.0114376)
Supplement: S2 Table — Summary of RNAseq coverage data using the Illumina genome analyzer. (DOCX) [file pone.0114376.s004.docx]

| **Table S2.** Summary of RNAseq coverage data using the Illumina genome analyzer | | | | |
| --- | --- | --- | --- | --- |
| Sample^a^ | Total number of reads | Filtered reads | Reads mapped to genome | Overall Alignment Rate (%) |
| CMCP6, HS (1) | 27,099,015 | 825,725 | 26,273,290 | 97.37 |
| CMCP6, HS (2) | 34,668,387 | 1,075,167 | 33,593,220 | 96.66 |
| YJ016, HS (1) | 45,576,619 | 1,403,108 | 44,173,511 | 98.16 |
| YJ016, HS (2) | 34,291,544 | 1,049,630 | 33,241,914 | 97.18 |
| CMCP6, ASW (1) | 36,508,024 | 1,080,730 | 35,427,294 | 97.57 |
| CMCP6, ASW (2) | 31,957,944 | 825,277 | 31,132,667 | 96.74 |
| YJ016, ASW (1) | 45,245,390 | 1,374,713 | 43,870,677 | 95.41 |
| YJ016, ASW (2) | 17,022,254 | 435,673 | 16,586,581 | 96.14 |

a - Indicated are the strain of *V. vulnificus*, incubation medium (human serum or artificial seawater), and biological replicate (1 or 2)
