# Supplementary material for: Transcriptome Sequencing Reveals the Virulence and Environmental Genetic Programs of Vibrio vulnificus Exposed to Host and Estuarine Conditions
Source: PLoS One. 2014 Dec 9;9(12):e114376. doi: 10.1371/journal.pone.0114376 (PMC4260858; doi:10.1371/journal.pone.0114376)
Supplement: S3 Table — Primers designed for this study. (DOCX) [file pone.0114376.s005.docx]

| **Table S3.** Primers designed for this study | | | | |
| --- | --- | --- | --- | --- |
| Gene annotation | Primer target^a^ | Sequence (5’ – 3’) | Product size (bp) | T_m_ (°C) |
| Glyceraldehyde phosphate dehydrogenase | *gapdh* F | TGAAGGCGGTAACCTAATCG | 97 | 60 |
|  | *gapdh* R | TACGTCAACACCGATTGCAT |  |  |
| Anti-anti-sigma regulatory factor, RsbR | *rsbR* F | GGCTCAGAAACACCCCTGAA | 162 | 60 |
|  | *rsbR* R | CACGGGCATGAATCTCTCCA |  |  |
| Negative regulator of sigma B, RsbS | *rsbS* F | GACCAGAGCAAAAGGGGTCA | 87 | 60 |
|  | *rsbS* R | TTCACGACATCGAGTAGGCG |  |  |
| Anti-sigma B regulatory factor, RsbT | *rsbT* F | GTACGAGATAAAGGGCCGG | 158 | 60 |
|  | *rsbT* R | GCAACAATCGTGGTGCCTTT |  |  |
| Negative regulator of sigma B activity, serine phosphatase, RsbU | *rsbU* F | TGCTGAGAAGTGGGTGTGAC | 80 | 55 |
|  | *rsbU* R | TCGCCAGAGACATATTCGCC |  |  |
| Downstream two-component sensor protein | TCSP F | GGAAGAGGCGGTTGAAGTGA | 128 | 60 |
|  | TCSP R | CATGCTGCTTGGGGGTAAGA |  |  |
